# Book Reviews

**Published:** 1903-04

**Authors:** 


Therapeutics of Dry Hot Air. By Clarence Edward Skinner, M. D., LL. D., New Haven, Conn., Professor of Thermotherapy in the New York School of Physical Therapeutics; Editor of the Department of Thermotherapy in the Journal of Advanced Therapeutics; Physician in charge of the Newhope Hot Air Sanitarium, New Haven, Conn. Octavo, pp. 200. Illustrated. New York: A. L. Chatterton & Co. 1903. [Price, $2.00.]

The therapeutic use of dry superheated air has been discussed heretofore in medical societies and journals, but this volume is the first book to treat exclusively of the subject. It records whatever is considered of importance relating to this method of treating certain diseases amenable to the influence of dry hot air. In the first chapter, the necessary apparatus is described; in the second, the physiological action of the remedy is discussed; and in the third, the technics of treatment are presented.

In the remaining chapters up to the twentieth, the various diseases in which the remedy is applicable are considered; and in the final chapter the author presents his conclusions, which offer hopefulness in the future for a further and more general application of the hot air treatment. He expresses the belief that hot air therapeusis is yet in its infancy, and then indicates the field of its future possibilities, citing diseases in which it presents prospects of amelioration or cure.

It is a well printed book, the paper is excellent, the engravings are good, and it deserves critical examination and application.

THE PRACTICAL MEDICINE SERIES OF YEAR BOOKS. Comprising ten volumes on the year's progress in Medicine and Surgery. Issued monthly under the general editorial charge of GUSTAVUS P. HEAD, M. D., Professor of Laryngology and Rhinology in the Chicago Post-Graduate Medical School. Volume II. General Surgery. Edited by John B. Murphy, M. D., Professor of Surgery, Northwestern University Medical School. Chicago: The Year Book Publishers. 1902. [Price, $2.00; entire series, $7.50.]

This volume is much larger than its predecessor of last year, embracing the same subject. This will enable the searcher to obtain sufficient details, for the most part, without consulting the original articles. Dr. Murphy, being an experienced surgeon, is admirably fitted for the work set before him, and it is difficult to see how it could have been performed in a more satisfactory manner. He has gleaned all the practical material from the surgical literature of the year, and served it up in a fashion that can be utilised with great dispatch and comfort.

Particular attention is paid to what has been accomplished in some of the newer fields of surgery, such as, for example, the surgery of the pancreas, the kidney, and the vascular and nervous systems. Dr. Murphy's operation for fixation of movable kidney will prove of great interest in this relation, and it may be pronounced as the simplest and most efficient of the several operations proposed for this condition. It is well described and illustrated in this book.

Many other subjects of interest and importance are dealt with by the editor, and it will be found to be a compilation of great helpfulness to the country surgeon in particular. It is an index of everything of value that has been published during the period which it covers.

---

BIOGRAPHIC CLINICS. The Origin of the Ill Health of DeQuincey, Carlyle, Darwin, Huxley and Browning. By GEORGE M. GOULD, M. D., Editor of American Medicine; Author of "An Illustrated Dictionary of Medicine." Duodecimo, pp. 223. Philadelphia: P. Blakiston's Son & Co. 1903. [Price, $1.00.]

A new medical literature is being constructed. We are to go back into the past, exhume the dead, hold autopsies on their remains, and from the examination construct a new pathology. At least that would seem to be the inference from reading this book.

It contains much of historical interest and is a clever study of psychologic ophthalmology. Not only will the ophthalmologist be interested in reading it, but so will those who have been entertained by the writings of the five men who form the subject of this post-mortem clinico-biographic refraction.

To assert that the author has discovered the cause of the ill health of these men of learning with absolute certainty, is more than can be determined with the data at hand, but it is quite probable that Dr. Gould has reached a point in the study that justifies the claim that eyestrain contributed to the conditions from which they suffered. At all events, the book should be read by all interested in the line of research which it pursues. It is a handsome piece of bookmaking.

---

BOOK ON THE PHYSICIAN HIMSELF, AND THINGS THAT CONCERN HIS REPUTATION AND SUCCESS. By D. W. CATHELL, M. D. The Twentieth Century Edition. Eleventh edition, revised and enlarged by the author and his son, William T. Cathell, A. M., M. D. Pp. 411, royal Octavo. Philadelphia: F. A. Davis Co. 1903. [Cloth, $2.50 net, delivered.]

The popularity of this book is unquestioned. It has passed to the age of maturity, to which its preceding ten editions testify, and has become part of the literature that every physician feels must be found in his library. It is a book full of good features, of good advice and of good intent, even though it contains much that is commonplace, and despite the fact that its literary style is, here and there, of questionable form.

Cathell gives excellent advice about writing for the medical press, and especially when he instructs that the title of an article should give some indication of its contents, he hits a solid nail squarely on the head. "Two cases from my note book," "A case of interest" and such titles, are about as silly as they are sophomoric.

This issue far excels its predecessors in letter press, paper and binding and is, in comparison, well entitled to its name—Twentieth Century Edition.

---

THE DEVELOPMENT OF THE HUMAN BODY. A Manual of Human Embryology. By J. PLAYFAIR MCMURRICH, A. M., Ph. D., Professor of Anatomy in the University of Michigan. Duodecimo, pp. 573. With 270 illustrations. Philadelphia: P. Blakiston's Son & Co. 1902. [Price, $3.00.]

This book furnishes the student who is inclined to ascertain reasons for certain accepted anatomical facts with the opportunity for interesting studies. The importance of beginning at the foundation in the investigation of any subject need not be debated, least of all when it pertains to anatomy.

A knowledge of embryology is becoming quite as necessary, as any branch of the anatomical field. The mysterious development of the human body should be closely investigated in order to appreciate the facts that a study of gross anatomy furnishes.

Professor McMurrich presents the subject in two parts, the first dealing with general development; and the second with organogeny. The five chapters in the first part begin with the spermatozoön and carry the development to and including the fetal membranes. The second part deals with the development of the organs, and also with post-natal development. While it is a concisely written work it yet preserves the charm of good English, and will prove a great aid to the student of anatomy in obtaining a better understanding of the genesis and development of the human body.

INTERNATIONAL CLINICS. A Quarterly of Illustrated Clinical Lectures and especially prepared Articles on Medicine, Neurology, Surgery, Therapeutics, Obstetrics, Pediatrics, Pathology, Dermatology, Diseases of the Eye, Ear, Nose, and Throat, and other Topics of Interest to Students and Practitioners by leading Members of the Medical Profession throughout the World. Edited by HENRY W. CATTELL, A. M., M. D., Philadelphia, with the Collaboration of John B. Murphy, M. D., Chicago; Alexander D. Blackader, M. D., Montreal; H. C. Wood, M. D., Philadelphia; T. M. Rotch, M. D., Boston; E. Landolt, M. D., Paris; Thomas G. Morton, M. D., Philadelphia; James J. Walsh, M. D., New York; J. W. Ballantyne, M. D., Edinburgh, and John Harold, M. D., London, with Regular Correspondents in Montreal, London, Paris, Leipsic, and Vienna. Volume IV. Twelfth series. Philadelphia and London. J. B. Lippincott Co. [Cloth, $2.00.]

The subjects embraced in this volume are therapeutics, five articles; medicine, seven articles; neurology, four articles; surgery, five articles; dermatology, one article; ophthalmology, one article; biographical sketches, two; and a monograph on the blood in health and disease.

One of the most interesting, if not instructive, papers in the book is the first one, by Dr. Charles Fox Gardiner, of Colorado Springs, entitled, The sanatory tent and its use in the treatment of pulmonary tuberculosis. It sets forth the value of out-door air in this disease, and emphasises the importance of its continuous employment during the entire twenty-four hours of each day. This can only be done by establishing the patient in a tent, and Dr. Gardiner has devised such a domicile, which not only meets the requirements but is comfortable and home-like. The article should be studied by every physician who treats this class of invalids.

The volume is filled with excellent papers on important subjects and indicates the popularity of this kind of medical literature. The steady improvement in the quality of material has been noted in these columns, and a standard has been reached leaving little to be desired.

---

A MANUAL OF DISSECTION AND PRACTICAL ANATOMY. Founded on Gray and Gerrish. By WILLIAM T. ECKLEY, M. D., Professor of Anatomy in the Medical and Dental Departments of the University of Illinois; the Chicago School of Anatomy and Physiology; the Chicago Clinical School and West Side Training Schools for Nurses. And CORINNE B. ECKLEY, Demonstrator of Anatomy in the Medical and Dental Departments of the University of Illinois. Large 8vo, pp. 414. Illustrated. Philadelphia and New York: Lea Brothers & Co. 1903. [Price, $3.50 net.]

The student of anatomy needs always a convenient manual for the laboratory. Heretofore most of such have been too condensed; whereas, the anatomical treatise is too elaborate, too cumbersome. The Eckleys have supplied the intermediate book in size and other points which go to supply the deficiency. The structures of the body have been identified with faithful accuracy and every detail of approach has been outlined with studious care.

The preference of students for the anatomical works of *Gray* and *Gerrish* has been catered to, by obtaining from the publishers of these books a large series of illustrations that never have been excelled for accuracy of detail or artistic merit. Of a total of 220 engravings, 116 are in colors, which, considering the size of the book, affords a wealth of illustration that in quality and quantity is unsurpassed.

The work besides being a guide for the dissector, is also a regional anatomy for the surgeon or prosector,—a combination of usefulness that has never been more carefully worked out. It is a work that deserves commendation from every viewpoint.

---

REGIONAL MINOR SURGERY. By GEORGE GRAY VAN SCHAICK, M. D., Attending Surgeon, French Hospital, New York. Duodecimo, 226 pages. Illustrated. New York: International Journal of Surgery Co. 1902. [Price, $1.50.]

The many serviceable hints given in this book must necessarily prove of benefit to the general physician who, perforce, must practise minor surgery. The text shows the experience of a practical surgeon in its preparation, and the illustrations bespeak the same origin. A few errors in punctuation will no doubt be corrected in a future edition, but they in no way obscure the sense to the practised eye. The book is to be commended especially to the junior practitioner, but may prove a valuable possession to any physician called upon to do minor surgery.

---

THE MATTISON METHOD IN MORPHINISM. A Modern and Humane Treatment of the Morphine Disease. By J. B. MATTISON, M. D., Medical Director, Brooklyn Home for Narcotic Inebriates. Duodecimo, pp. 40. New York: E. B. Treat & Co. 1902. [Price, $1.00.]

This instructive brochure is the outcome of thirty years experience by the author in the treatment of morphine disease. It gives in detail his method of treatment which appeals, at once, to the judgment of every scientific physician. It is a disease that cannot be treated by the rule of thumb, but each particular case must receive such care and administration of remedies as experienced judgment dictates. It would be well for every physician interested in the subject of which it treats, to read this book with attention, because it contains useful scientific information upon a subject that appears to be growing in importance as time advances.

---

REPORT OF THE COMMISSIONER OF EDUCATION FOR THE YEAR 1900-1901. Volume I. Washington: Government Printing Office. 1902.

This volume contains much information of interest to the cause of education in general, as well as items and statistics relating to schools and colleges in particular,—that is, at home and abroad. It also contains a list of educational periodicals published in the United States, and an educational directory of names of state and city school principals and college presidents, which must prove of great convenience. But, most important or most interesting of all, it contains a history of the foundation of the Carnegie Institution, at Washington. The aims of this institution, as set forth in the trust deed of the donor are as follows:

1. To promote original research, paying great attention thereto as one of the most important of all departments.

2. To discover the exceptional man in every department of study whenever and wherever found, inside or outside of schools, and enable him to make the work for which he seems specially designed his life work.

3. To increase facilities for higher education.

4. To increase the efficiency of the universities and other institutions of learning throughout the country, by utilising and adding to their existing facilities and aiding teachers in the various institutions for experimental and other work in these institutions as far as advisable.

5. To enable such students as may find Washington the best point for their special studies to enjoy the advantages of the museums, libraries, laboratories, observatory, meteorological, piscicultural, and forestry schools, and kindred institutions of the several departments of the government.

6. To ensure the prompt publication and distribution of the results of scientific investigation, a field considered highly important.

---

Memoranda on Poisons. By Thomas Hawkes Tanner, M.D., F.L.S. Ninth revised edition, by Henry Leffmann, A.M., M.D., Professor of Chemistry in the Woman's Medical College of Pennsylvania. Philadelphia: P. Blakiston's Son & Co. 1902. [Price, 75 cents.]

It is but little more than a year since the eighth edition of this useful little manual was noticed in these columns. It is important for every physician to keep his mind refreshed in regard to poisons and their antidotes. Nothing has ever been published that equals this little classic for the purpose indicated. In frequent editions revision is made so that the memoranda are kept abreast of the period; and, besides, it can be relied upon as a safe guide, and is of easy and quick reference.

---

Lea's Series of Pocket Textbooks. A Pocket Textbook of Anatomy. By William H. Rockwell, Jr., M. D., Assistant Demonstrator of Anatomy, College of Physicians, Columbia University, New York. In one 12mo volume of 600 pages, with 70 illustrations. Edited by Bern B. Gallaudet, M. D. Philadelphia and New York; Lea Brothers & Co. 1903. [Price: cloth, $2.25 net; limp leather, $2.75 net.]

A condensation of anatomy is of value oftentimes, not only to the student, but to physician, surgeon, and even others who need either quick reference or extended general examination of some portion of the topic. This work has been prepared by an anatomist of experience, and presumably one who knows just what is most needed by those for whom the book is especially designed. It is based upon *Gray*, which is easily the most popular anatomy with students. It is an addition to the pocket textbook series, of which it forms a part, and is a fine specimen of medical bookmaking.

TRANSACTIONS OF THE STATE MEDICAL ASSOCIATION OF TEXAS. Thirty-fourth Annual Session, held at Dallas, Texas, May 6-9, 1902. H. A. WEST, M. D., Secretary. Austin: Von Boeckmann, Schutze & Co. 1902.

The physicians of Texas are entitled to congratulations on account of the splendid state medical association which they maintain, and the excellent report made of its proceedings in the annual volume of transactions which is issued under the direction of its accomplished secretary.

At the meeting reported in this book considerable time was spent in considering the majority and minority reports of the committee on reorganisation, the result of the discussion being to carry it over for a year, and the committee continued to finish its work.

The papers on scientific medicine are of great value and the volume, consisting of 587 pages, does credit to the society and the secretary, whose labors are especially arduous and ought to be appreciated, as, no doubt, they are.

---

TRANSACTIONS OF THE MEDICAL ASSOCIATION OF THE STATE OF ALABAMA. Annual Meeting, held at Birmingham, April 15-18, 1902. G. P. WALLER, M. D., Secretary. Montgomery: Brown Printing Co. 1902.

This sterling society, one of the most progressive and best organised in the south, presents its transactions in excellent form and with careful preparation. It holds its sessions during four days, including three evening sessions,—an example that might well be followed by the Medical Society of the State of New York.

The anniversary address of the presiding officer is called "the president's message," and is a paper which sets forth the condition of medical affairs in the state. The book contains reports from the officers, standing committees, the State Examining Board and the State Board of Health; and then follow scientific papers regular and volunteer, together with a list of the physicians in the state grouped by counties.

It is a book full of interest to every physician in the state of Alabama, and many portions of it are interesting to others.

---

TRANSACTIONS OF THE STATE MEDICAL SOCIETY OF WISCONSIN. Fifty-sixth Annual Meeting, held at Milwaukee, June 4-6, 1902. CHARLES S. SHELDON, M. D., Secretary. Madison: State Journal Printing Co. 1902.

This well-organised and disciplined medical society again presents the proceedings of its annual meeting in good form. The volume likewise is full of good material.

The president's address is devoted in large measure to a discussion of the reorganisation of the society, on the plan proposed by the American Medical Association. He opposed the report of the committee, the society agreed with the president and the subject went over another year, the committee being continued.

The scientific papers are of a superior order, one of the best being by Dr. Scollard, relating to the diagnosis of fetal position and presentation by external examination. There are, however, half a dozen or more excellent articles of general or special interest, the whole book being of unusual quality.

